# Lithiation-Dependent Micromechanical Response of Amorphous and Crystalline MoO_3_ Thin-Film Cathodes on Al Current Collectors

**DOI:** 10.1021/acsomega.6c00534

**Published:** 2026-06-25

**Authors:** Dávid Ugi, Lakshmi Shiva Shankar, G. Z. Radnóczi, Péter Dusán Ispánovity, Robert Kun

**Affiliations:** † HUN-REN Research Centre for Natural Sciences, Institute of Materials and Environmental Chemistry, Magyar Tudósok Körútja 2, 1117 Budapest, Hungary; ‡ ELTE Eötvös Loránd University, Department of Materials Physics, Pázmány Péter Sétány 1/a, 1117 Budapest, Hungary; § Széchenyi István University, Zalaegerszeg Innovation Park, Dr. Michelberger Pál út 3, H-8900 Zalaegerszeg, Hungary; ∥ HUN-REN Centre for Energy Research, Konkoly-Thege M. u. 29-33, Budapest H-1121, Hungary; ⊥ HUN-REN Wigner Research Centre for Physics, Institute for Solid State Physics and Optics, Konkoly T. út 29-33, Budapest 1525, Hungary; # Széchenyi István University, Sustainability Competence Centre, Egyetem square 1, H-9026 Győr, Hungary

## Abstract

In this study, the mechanical response of MoO_3_ thin-film cathodes deposited on aluminum substrates was systematically investigated using nanoindentation techniques under an inert atmosphere. Both amorphous and crystalline phases were examined across non-, partially, and fully lithiated states to elucidate the influence of lithium intercalation on elastic and plastic behavior. A range of indenter geometries, including spherical and Berkovich tips, were employed to extract plastic, elastic, and interfacial properties. The elasticity increased with lithium content, with partially lithiated systems exhibiting the highest values. Residual indentation depths were lowest for partially lithiated samples, indicating a distinct mechanical regime compared to both non- and fully lithiated states. The amorphous phase demonstrated higher stiffness, with deformation-induced cracks confined within the layer, while the crystalline phase accommodated deformation more uniformly via grain boundary sliding. The mechanical response in the crystalline phase suggests a significant role of grain-boundary-mediated deformation mechanisms. Furthermore, no degradation in layer adhesion was observed with increasing lithium content, indicating a mechanically stable interface across all lithiation states. These findings provide new insights into the mechanical integrity of cathode–current collector systems in solid-state lithium-ion batteries and underscore the critical role of intercalation state, structural phase, and microstructural pathways in determining mechanical performance.

## Graphical abstract

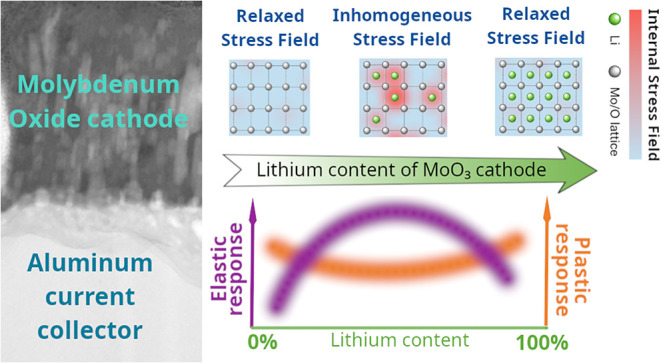

## Introduction

In recent decades, the demand for portable power has steadily increased due to the increasing need for mobile handheld devices, electric vehicles, aerospace applications, and more. The usage conditions for these batteries can vary based on their application. Thin metal oxide films have long been recognized as promising materials for battery electrodes, since they have sufficient conditions such as manufacturing costs, safety, and autonomy with reduced weight and size. It is possible to lower the thickness of thin films to a point where the electrochemical behavior is not significantly influenced by the electrical conductivity.^1,2^

Regarding the battery performance, the phase and the microstructure of the electrode also play a critical role.^2–6^ Direct current (DC) sputtering is an attractive method for the preparation of thin-film metals due to the target’s high conductivity. Sputtered metal oxide thin layers are rather amorphous in nature, but they could be transformed into a polycrystalline form by means of heat treatment.^3,7^ Nevertheless, temperature annealing often results in interphase formation.^8^

The characterization of the mechanical properties of the substrate-thin film composite, including interlayer effects, is a highly challenging and currently ongoing process. Unfortunately, there is still no uniformly usable experimental method for this purpose; however, several methods that work well for a given system have been developed. For example, cantilever and notched-cantilever bending,^9,10^ scratch tests,^11,12^ crack propagation,^13^ tension (via layer crack density),^14^ shear tests (pure and mixed),^15^ or cross-sectional indentation tests.^16^

Nanoindentation tests on substrate–thin film composites have also proven to be a useful tool for mechanical characterization. For this purpose, the individual and related mechanical parameters of the indenter, film, and substrate are required.^17–20^ In the hard layer–soft substrate systems investigated in our study, previous works have shown that when the ratio of indentation depth to film thickness exceeds *h*/*t* > 0.35, the response begins to reflect the mechanical contribution of the substrate as well. Moreover, regarding the layer–substrate coupling characteristics, earlier studies concluded that indentation-based methods can only provide qualitative insights.^21^

### Molybdenum Oxide Thin Layer

The mechanical properties of all-solid-state battery (ASSB) cathodes are strongly dependent on their states of lithiation. A thorough understanding of these properties is essential as the extent of such changes fundamentally determines the lifetime of these batteries. Accordingly, increasing attention is being devoted to this topic with investigations now relying on advanced characterization methods. ASSB cathodes composed of transition-metal oxides exhibit high energy density,^22^ which motivated the selection of such materials for the present study. Literature reports have shown that the performance of these cathodes is significantly influenced by their precise microstructure, including the crystallographic orientation, grain size, and dislocation structure.^6,23^

To investigate the microscopic effects of lithium intercalation in cathode materials, Liu et al. developed a nanobattery within a transmission electron microscope (TEM), enabling real-time and atomic-scale observation of the charging and discharging processes. Their findings revealed an unexpectedly pronounced anisotropic volume expansion due to lithium intercalation as well as cracking induced by stress concentrations.^24,25^

Transition-metal oxides are widely investigated as cathode materials due to their ability to accommodate lithium through intercalation reactions accompanied by structural and electronic changes.^22^ In such systems, lithium insertion leads to lattice distortion, internal stress development, and modification of bonding environments, which can directly influence mechanical behavior.

Amorphous and crystalline oxide phases exhibit distinct lithium storage characteristics. Amorphous metal oxides often provide a higher density of accessible lithium insertion sites and enhanced structural adaptability during lithiation due to their disordered structure.^2,5^ Molybdenum trioxide (MoO_3_) is a well-established lithium intercalation material that forms LixMoO_3_ phases upon lithiation, as demonstrated by spectroscopic studies.^26^ These characteristics make MoO_3_ a suitable model system for investigating the coupling among lithium intercalation, microstructure, and mechanical response.

### Lithium Intercalation

The lifetimes of the cathode and its interfaces are largely determined by their structural stability and mechanical integrity. To assess this, an atomistic understanding of lithium intercalation within the cathode material is essential. The application of crystalline forms of metal oxides as cathode materials has been extensively studied. In such systems, lithium is inserted into crystallographic sites within the cathode during redox reactions, a process that induces mechanical stress, lattice defects, and structural and chemical changes. The lattice sites of lithium incorporation are associated with specific crystallographic features that can lead to anisotropic and inhomogeneous structural changes during intercalation.^6,23,26–29^

Electrode materials have traditionally been predominantly crystalline; however, amorphous materials are gradually emerging as promising alternatives. Although numerous long-range-disordered materials have been proposed as cathodes for ASSBs, their classification and chemo-physical characterization remain incomplete.^30^ Amorphous metal oxides (AMOs), such as the system investigated in this study, have attracted significant attention in recent years because of their intrinsic isotropy, the absence of grain boundaries, and distinct defect distributions.^2^ Previous studies have demonstrated that, even in AMOs, lithium intercalation and the associated redox reactions are bulk processes rather than surface-limited phenomena.^31^

In the case of amorphous TiO_2_, intercalated Li^+^ ions are located within distorted oxygen polyhedron lattice sites analogous to the octahedral configurations found in the crystalline phase. These polyhedral environments, although distorted, are also generally present in the amorphous structure of AMOs, suggesting that the fundamental lithium storage mechanisms may be similar across different phases of a given material. The required Li^+^ diffusion is believed to proceed along pathways that connect these vacant polyhedral sites.^32,33^ While the exact lithium diffusion mechanism in the amorphous phase is not yet fully understood, it is known that such disordered structures may offer a higher density of intercalation sites, shorter diffusion paths, and enhanced accommodation of structural strain during the intercalation process.^2,34^ In the present work, lithiation states are defined based on controlled electrochemical conditions, enabling a systematic comparison of mechanical response at different degrees of lithium insertion.

## Materials and Methods

### Sample Preparation

In order to investigate the mechanical properties of multilayer structures characteristic of ASSBs, we deviated from the conventional substrates typically used for PVD-deposited layers. Instead, we employed an aluminum substrate (also suitable as a current collector material) with a diameter in the centimeter range and a thickness of several millimeters. The substrate was polished using 50 nm AlOx polishing powder, resulting in a surface with sufficient smoothness for thin film deposition. To reduce the internal stresses and crystal defects accumulated in the substrates—and on the surface caused by the mechanical polishing—a heat treatment was applied: 300 °C for 3 h, with a heating rate of 10 °C/min.

The deposition of the molybdenum oxide (MoO_3_) layer onto the substrate was carried out based on our previous work.^3^ A pure Mo target was used with a magnetron power of 200 W in a controlled atmosphere. The gas flow consisted of 60 sccm Ar and 20 sccm O_2_, establishing a deposition pressure of 8.7 ± 0.1 × 10^–3^ mbar.

The resulting MoO_3_ thin film was confirmed to be amorphous by previous findings, and the crystallization was achieved using the same heat treatment as before^3^ (300 °C, 3 h, 10 °C/min). To minimize the influence of thermal treatment on the substrate during the crystallization of the layer, the same 3 h heat treatment was repeated on the substrates that would later support the amorphous layers, prior to deposition. This approach was intended to ensure that the microstructure of the substrate remains unaffected by the subsequent structural changes in the MoO_3_ thin film.

### Nanoindentation

In situ indentation experiments were performed inside a MB200 Braun glovebox under an argon atmosphere, with oxygen and water contents below 1 ppm. A custom-built nanoindenter was used, which operated without a load or strain feedback loop. Instead of the traditional control mode, a constant platen velocity was applied during the tests to define the average strain rate, as in a previous study.^35,36^ The precision of indentation depth and load measurements was approximately 1 nm and 1 μN, respectively.

Indentation experiments were conducted on molybdenum oxide layers in both crystalline (heat-treated, Ht) and amorphous (as-deposited, Ad) phases, as well as on bare substrates. To investigate both plastic and elastic properties, three different indenter tips were employed: a sharp Berkovich tip and spherical tips with radii of 2 μm and 10 μm. These tips were used interchangeably within the same instrument by replacing the indenter tip assembly, ensuring identical instrument conditions for all measurements. The indentation procedure for both Ht and Ad layers was identical, using a constant spring velocity of 25 N m/s, which corresponds to the average penetration rate. For each indenter tip, two sets of three indentations were performed.

On the basis of previous measurements, to evaluate the effect of the substrate and interlayer interaction on layer properties, three indentations were carried out, with a maximum load of 4 mN, followed by a 20 s hold period and unloading at the same rate. This was followed by three additional indentations with a maximum load of 6 mN, also with a 20 s holding time.

By considering the entire indentation load–displacement curve, a rapid estimation of the elastic response of the system can be obtained. According to the Oliver-Pharr methodology,^37^ the system’s effective elastic modulus is given by *E*_r_ *=* √π/2 × *S*/√*A*, where *S* is the slope of the linear fit applied to the initial portion of the unloading curve and *A­(h*_c_ *)* is the projected contact area at maximum load. For a Berkovich tip, *A*_b_ *=* 24.5 *h*_c_^2^, while for a spherical indenter, *A*_s_ *=* π*Rh*_c_, where *R* is the tip radius.^39^ Since the same instrument, calibration, maximum load, and indenter tip material were used for all measurements, systematic contributions from the indenter modulus and instrument compliance remain constant across experiments. Therefore, for comparative purposes, a tip-geometry-dependent elastic response parameter can be defined as ERP *= S*/√*A*_geo_, where *A*_geo_ depends on the indenter tip geometry.

To investigate the mechanical effects associated with the operation of an Assb, the indentation method described above (a total of 36 experiments) was applied to molybdenum oxide thin films in different states of lithiation. For both Ad and Ht samples, measurements were conducted not only in the unlithiated (0% state) condition but also in fully lithiated (100%) and partially lithiated (50%) states. In total, 108 indentations were analyzed.

### Electrochemical Characterization and Sample Preparation for Mechanical Testing

To investigate the effect of lithium intercalation on the mechanical response of MoO_3_ thin film cathodes, electrochemical cycling was performed before nanoindentation testing. Thin-film electrodes in both amorphous and crystalline phases were integrated into 2032-type coin cells, where controlled lithiation states were established through galvanostatic charge–discharge protocols. This approach enabled a direct correlation between electrochemical state-of-charge (SoC) and micromechanical properties across well-defined lithiation levels.

#### Coin Cell Assembly and Electrochemical Cycling Protocol

The MoO_3_-coated aluminum substrates (12 mm diameter) were assembled in an argon-filled glovebox (H_2_O/O_2_ < 0.5 ppm) using a standard CR2032 cell setup. Metallic lithium foil served as the counter and reference electrode, and Whatman Glass Fiber served as the separator. The electrolyte was composed of 1 M Lithium hexafluorophosphate (LiPF_6_) in a 1:1 v/v mixture of ethylene carbonate (EC) and dimethyl carbonate (DMC), commonly used in Li-ion cell testing due to its electrochemical stability. After drying the thin film electrodes under vacuum at 60 °C, each was placed in direct contact with the lithium counter electrode and wetted with ∼100 μL of electrolyte before sealing. No binder or conductive additives were used to isolate the intrinsic electrochemical and mechanical behavior of the active layer.

Galvanostatic charge–discharge cycling was carried out at a C/10 rate, defined with respect to the theoretical capacity of MoO_3_ (279 mAh g^–1^), using a Biologic VMP-300 potentiostat controlled by EC-Lab software. The first cycle was used to stabilize the electrode–electrolyte interface and form a solid electrolyte interphase (SEI), especially relevant for the amorphous samples. Representative first-cycle voltage–capacity curves for both amorphous and crystalline MoO_3_ thin film electrodes are provided in the Supporting Information (Figure S1).

The voltage–capacity curves exhibit a predominantly sloping profile during both lithiation and delithiation, without distinct voltage plateaus. This behavior is characteristic of solid-solution-type lithium insertion in nanostructured and amorphous transition-metal oxides, where lithium is accommodated over a distribution of energetically nonequivalent sites rather than via a well-defined two-phase reaction.

The measured capacities are relatively low (on the order of 10^–3^–10^–2^ mAh), which is expected due to the thin-film geometry and the absence of conductive additives or binders. In this configuration, the electrochemical measurements are not intended to evaluate battery performance but rather to establish well-controlled lithiation states for subsequent mechanical characterization. Subsequently, cells were discharged under controlled conditions to specific voltage cutoffs to yield three distinct lithiation states:

- 0% SoC (unlithiated): No electrochemical cycling applied; pristine state.
- 50% SoC (partially lithiated): Discharged to ∼1.9 V vs Li/Li^+^, providing an intermediate electrochemical state.
- 100% SoC (fully lithiated):Discharged to 1.5 V vs Li/Li^+^, corresponding to the voltage cutoff applied in this study.

After reaching the desired SoC, cells were disassembled in an inert atmosphere, and the cathodes were thoroughly rinsed with dimethyl carbonate (DMC) to remove the electrolyte residue. These films were dried under a dynamic vacuum before proceeding to in situ mechanical characterization via nanoindentation.

The decision to perform controlled lithiation stems directly from the central hypothesis of the study: the mechanical response of cathode thin films is strongly coupled to their electrochemical states. Lithium intercalation induces volumetric expansion, generates internal stresses, and alters bonding environments, all of which significantly influence the elastic and plastic properties of the electrodes. By preparing the films at 0%, 50%, and 100% SoC, the study was designed to explore not only the limits of mechanical performance but also intermediate regimes where partially filled Li^+^ sites may act as pinning centers or defect stabilizers. As shown later in Figures 7 and 8, the partially lithiated samples exhibit the highest elastic response and the lowest residual indentation depths, indicating a distinct mechanical regime compared with the other states.

It should be noted that the lithiation states in this study are defined based on controlled electrochemical conditions (voltage cutoffs) rather than direct structural or chemical characterization. Although some variation is observed in the voltage–capacity response between samples, the applied galvanostatic protocol and defined voltage limits ensure consistent and well-controlled electrochemical states for comparative analysis. However, this does not provide direct insight into lithium distribution or phase evolution within the material, but rather defines controlled electrochemical states for subsequent mechanical characterization. Therefore, the mechanistic interpretations are based on the electrochemically defined states and corresponding mechanical response, rather than on direct structural verification.

### Electron Microscopy

Because of the extreme reactivity of our deposited layer upon lithium incorporation, electron microscopy analysis in the lithiated state proved to be challenging. Therefore, electron microscopy investigations were conducted on the nonlithiated samples. The surface morphology of the deposited layers was examined by using an FEI Quanta 3D dual-beam scanning electron microscope (SEM). Additional transmission electron microscopy (TEM) investigations were performed by using a Cs-corrected Themis-type electron microscope operating at 200 keV. These experiments were conducted on cross-sectional FIB-cut (Thermo Fisher Scios2) lamellae prepared from the indentation marks, targeting the local and interlayer high-resolution characterization.

## Discussion

### Substrate

Comparative measurements were also performed on the bare aluminum substrate. These tests were conducted on regions of the noncoated samples where, due to masking during deposition, no thin film was present. The general shape and features of the load–displacement curves remained consistent across regions with and without the thin film, aside from a few notable exceptions. These validate the successful substrate preparations. Also, in all cases, the presence of the thin film was associated with a general hardening effect, as their maximal penetrations at the same load values were significantly smaller (Figure 1, without, and Figure 8, with coating). For the indentations with a 2 μm spherical tip, all the curves showed characteristic serrations, stochastic stress drops, and strain bursts, as illustrated in Figure 1.

**Figure 1:**
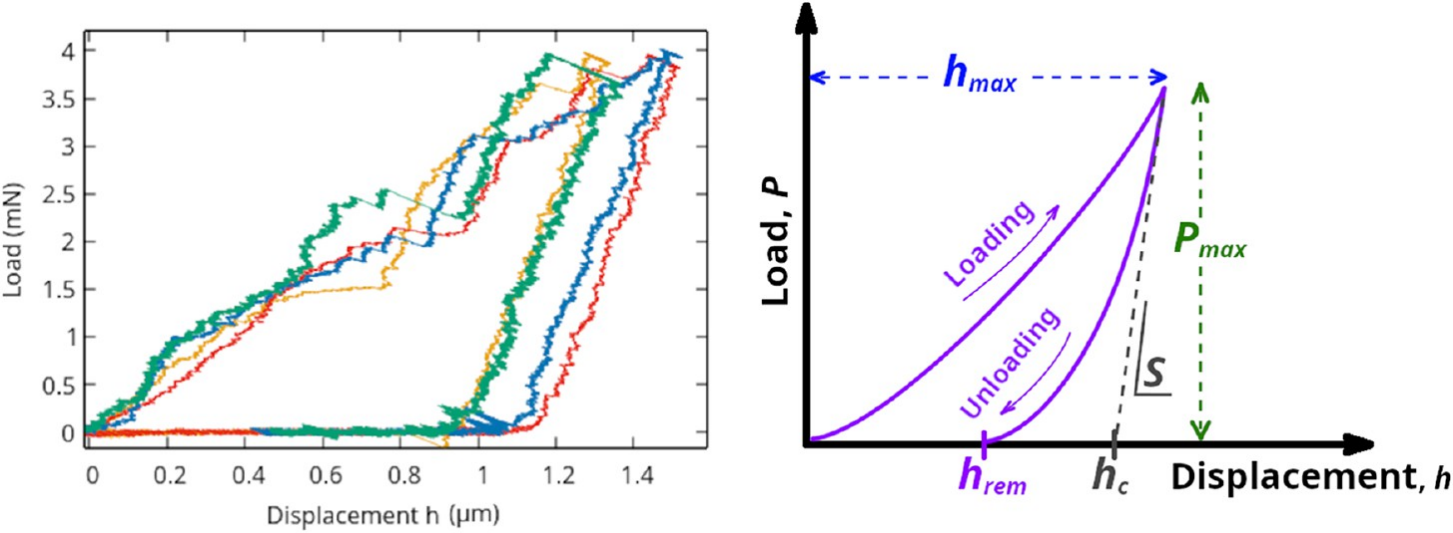
Left: Four load–displacement curves of the bare aluminum substrate measured with the 2 μm spherical indenter. Right: Drawing of an identical load–displacement curve with the applied parameters.

The substrate material response observed during indentation with the 2 μm radius spherical tip differed compared to those obtained using other indenter geometries. It is well known that, for crystalline materials, load–displacement curves obtained with spherical indenters can exhibit a stochastic character and depend on the radius of the indenter tip.^38,39^ The occurrence of these strain bursts is attributed to the sudden release of elastic energy accumulated beneath the indenter, triggered by cooperative dislocation motion. For such events to occur, both the geometry of the indenter and the dislocation density of the material—as well as external parameters such as the indenter control mode, strain rate, and temperature—must fall within a specific range.^40,41^

### Phase Transformation Effect Based on Electron Microscopy

In our previous work, we have already investigated the effect of phase transformation.^3^ While the present study primarily focuses on the mechanical effects of lithium intercalation, we have also made additional observations regarding phase transformation. In the case of nonlithiated layers, cracks induced by indentation in the amorphous phase confirm the brittle nature of the layer, whereas the pile-ups observed around the indentation patterns in the crystalline phase indicate a more plastic behavior (Figure 2). SEM observations revealed that even in regions unaffected by indentation, cracks appeared in the crystalline layers. These are most likely caused by volume changes associated with phase transformation.

**Figure 2:**
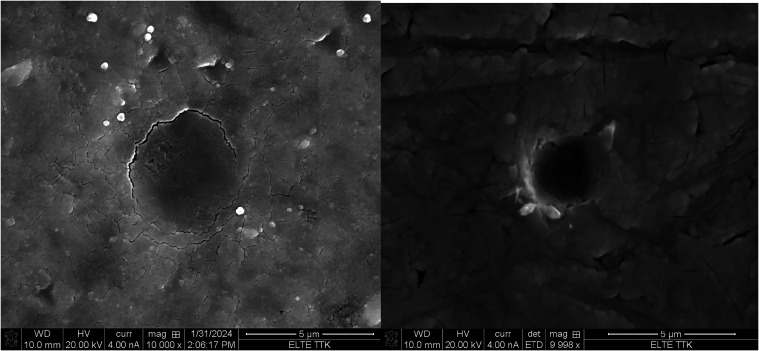
Indentation patterns of the 2 μm spherical tip. Left: amorphous; right: crystalline form.

Based on TEM images, the thickness of the deposited layer was examined locally. For the amorphous phase layer, using the method detailed in the Sample Preparation section, the thickness was determined to be 195 ± 4 nm, based on measurements taken at 20 different points. The same measurement conducted on the crystalline phase yielded a thickness of 175 ± 7 nm. The 12% difference between the two thickness values is significantly larger than the variation that could be attributed to deposition conditions (such as gas pressure, deposition duration, temperature, target-sample distance, or magnetron power). Therefore, this difference can reasonably be ascribed to the expected phase transformation, which also explains the cracks observed in regions distant from the indentation pattern only on the right side of Figure 2.

The TEM observations provide microstructural context for interpreting the indentation response, particularly with respect to phase-dependent deformation mechanisms. Cross-sectional TEM lamellae were prepared from the 10 μm spherical indentation patterns using FIB milling for both the amorphous (Figure 3) and crystalline (Figure 4) cases. In the amorphous layer, different types of cracks were observed based on TEM images. Platinum (Pt) deposited during the lamella preparation process was detected inside cracks indicated by black arrows on the last image of Figures 3 and 6, suggesting that the layer separates along cracks induced by deformation. Additionally, further cracks marked with purple arrows were observed; however, these do not disrupt the continuity of the layer, similarly to shear bands typically observed in amorphous materials.^42,43^ These latter cracks are located in the vicinity of the indentation imprint and are therefore attributed to the indentation process. In the crystalline phase layer, the microcracks observed in the amorphous phase were detected in much lower concentration, supporting that deformation occurs predominantly via dislocation activity and grain boundary sliding in this layer. However, Pt-containing cracks were also detected (Figure 5), similarly to the amorphous case. All observed cracks are oriented vertically, and the absence of cracks running parallel to the interlayer interface indicates a high-quality adhesion between the layer and the substrate (Figure 6).

**Figure 3:**
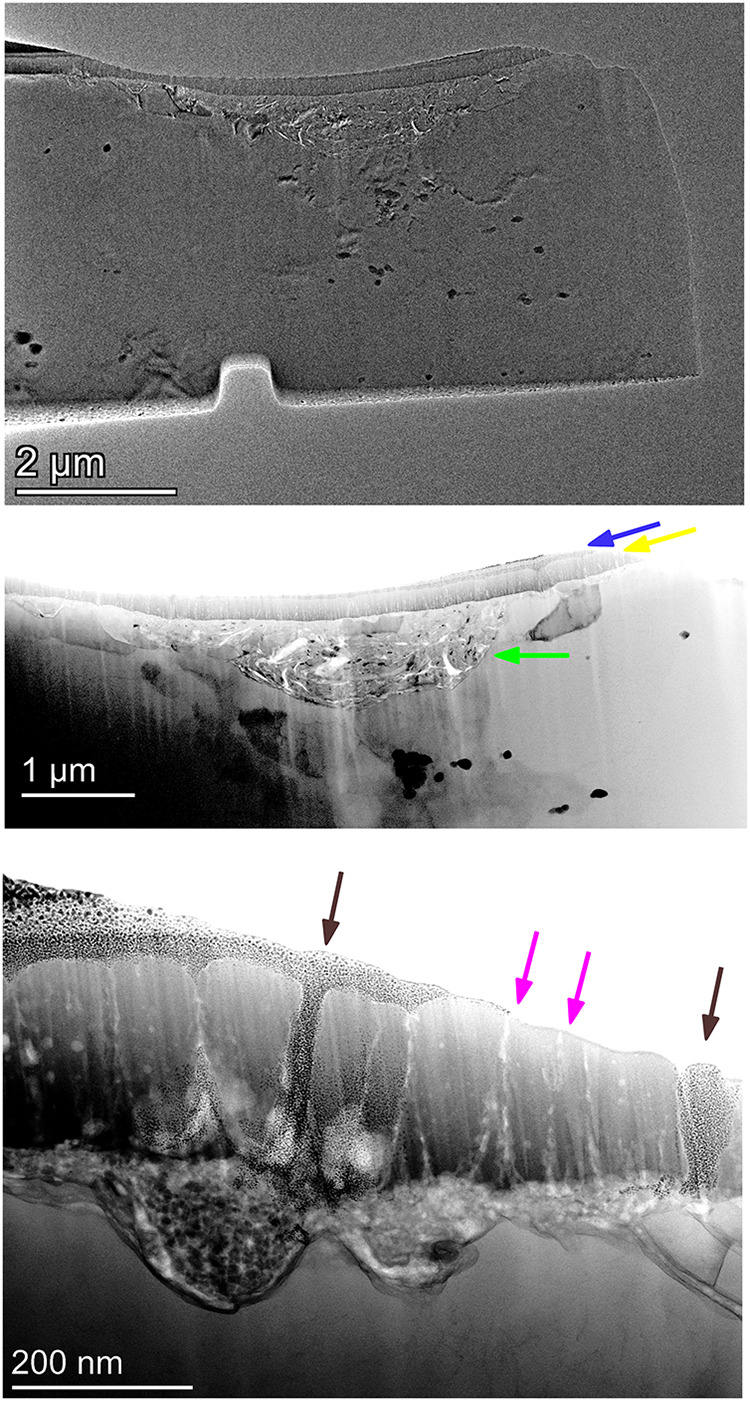
TEM images of a spherical 10 μm indentation pattern in the amorphous layer. The first image shows an overview of the indentation. The second image shows the details of the structure in bright field, while the last image shows cracks present in the layer after indentation. Blue arrow: protective platinum layer applied during cross-sectional lamella preparation; Yellow arrow: as-deposited amorphous molybdenum oxide layer; Green arrow: deformed region of the substrate; Purple arrows: vertical cracks through the deposited layer; Black arrows: cracks also visible in SEM images, indicating discontinuities in the layer.

**Figure 4:**
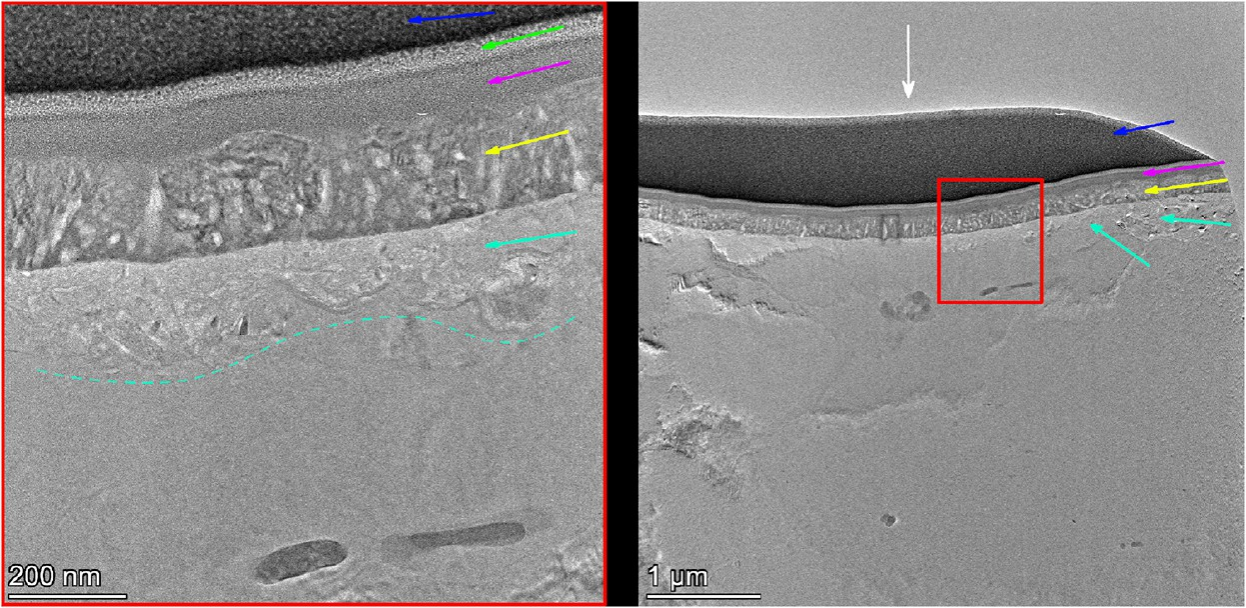
TEM image of a spherical 10 μm indentation pattern in the crystalline layer. The inset on the left shows a magnified view of the area indicated by the red rectangle. White arrow: loading direction; blue arrows: protective platinum layer deposited during cross-sectional lamella preparation by FIB; pink arrows: protective Pt layer deposited by electron beam; green arrow: ion beam-affected zone within the electron-deposited Pt layer; yellow arrows: heat-treated, polycrystalline molybdenum oxide layer; cyan arrows: deformed region of the substrate.

**Figure 5:**
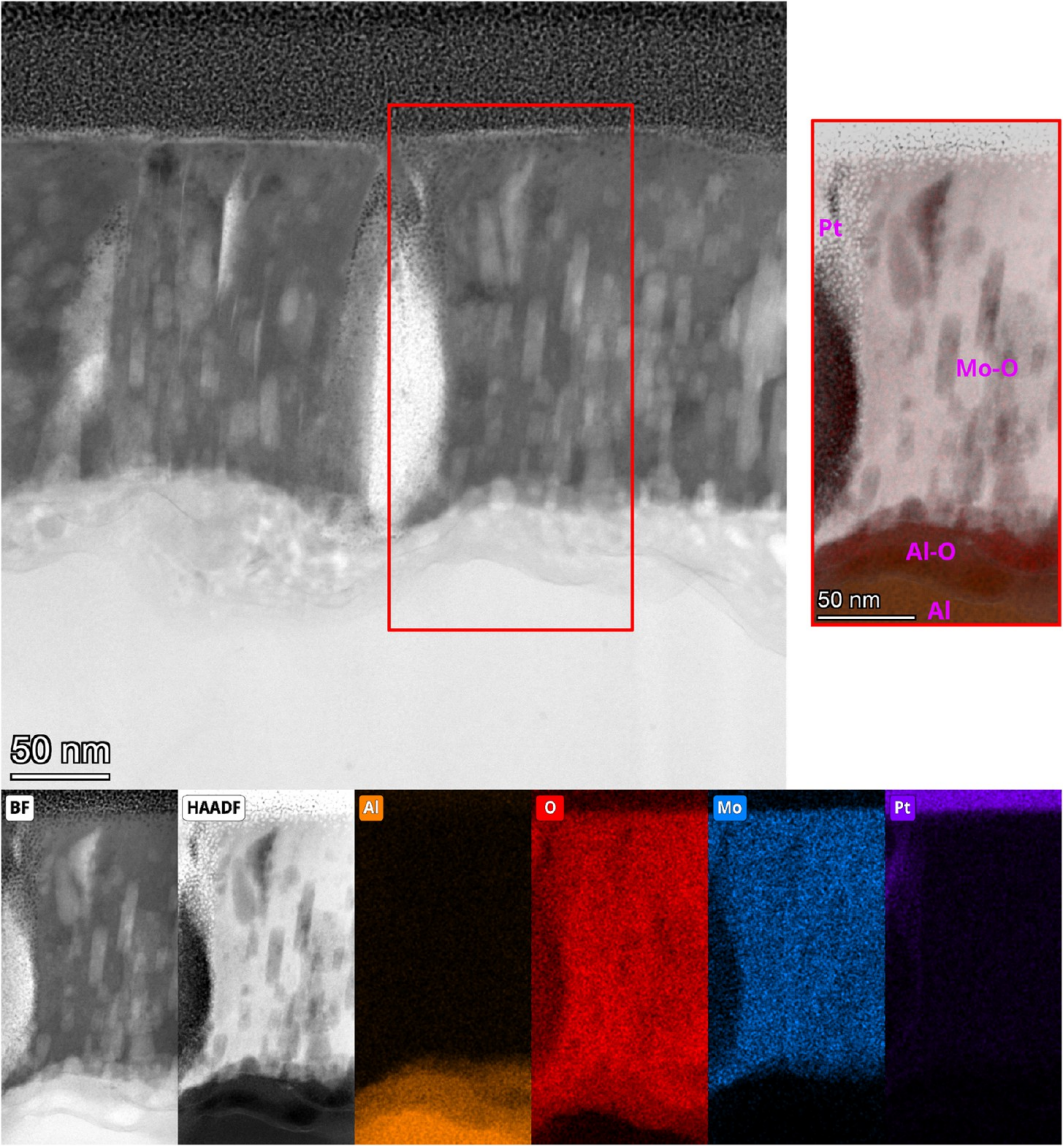
Magnified view of Figure 4 (crystalline) and elemental map of the red-marked region on the right side.

**Figure 6:**
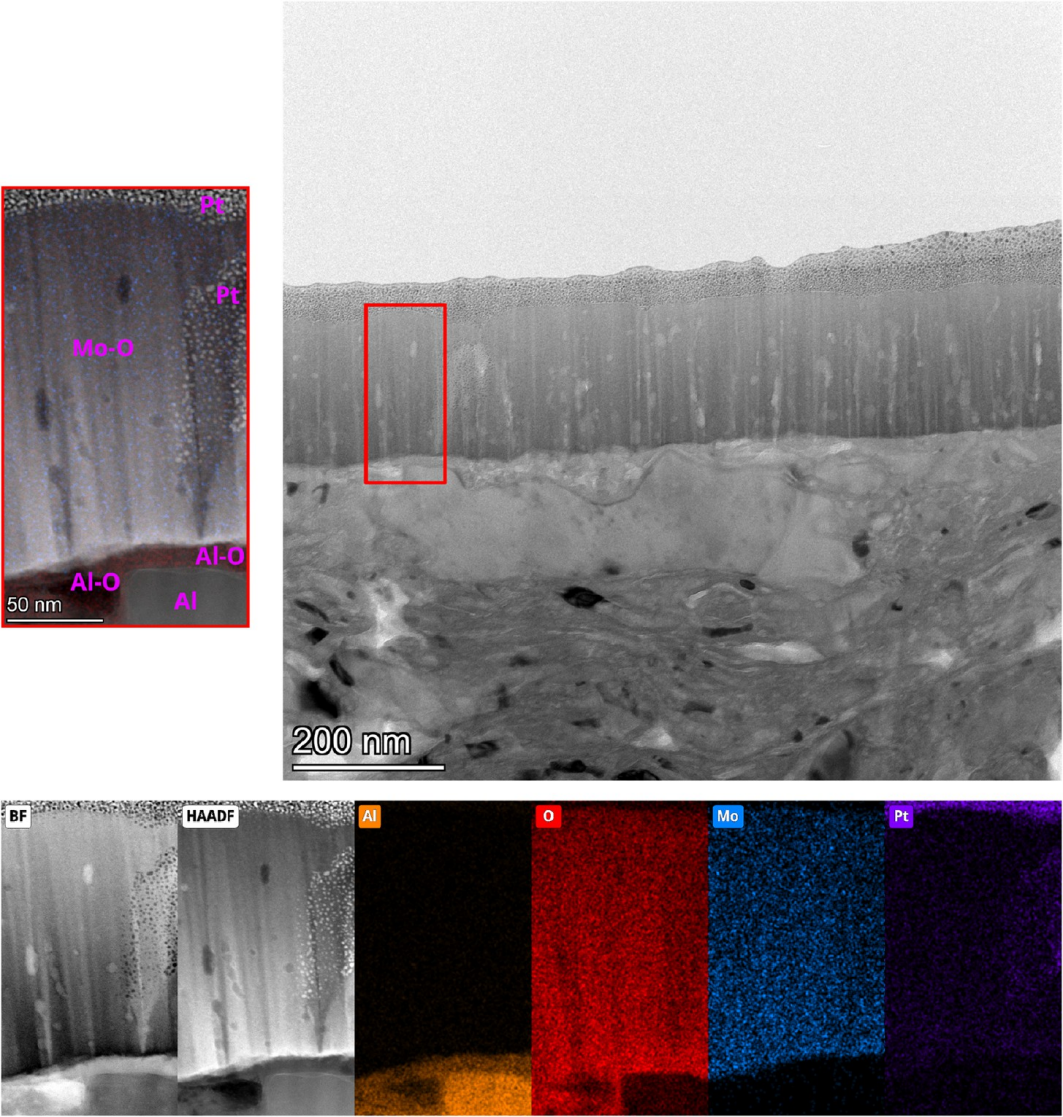
Magnified view of Figure 3 (amorphous) and elemental map of the red-marked region on the left side.

Chemical analysis also revealed that the deposited thin film had formed on the naturally occurring, well-adhering oxide layer of the aluminum substrate, with a measured thickness of 43 ± 13 nm, as shown in Figure 5. The most common oxide is amorphous alumina, which typically forms a 2–3 nm thick film upon atmospheric exposure of aluminum at room temperature.^44^ Given that the oxide layer identified in our study is significantly thicker than this, and considering that such layers can also be produced by reactive magnetron sputtering,^45^ we propose that a chemical reaction occurred within the native Al oxide layer during Mo oxide film deposition or subsequent heat treatment. As the oxide interfaces appear stable and well-defined (Figure 5), this observation will be referenced only briefly hereafter, since the adhesion of the investigated Mo oxide film is primarily governed by the properties of the underlying Al oxide layer.

To examine whether the additional heat treatment—aimed at crystallizing the deposited thin layer—has an effect on the substrate’s native oxide layer, we also performed the chemical analysis described in the previous paragraph for the amorphous thin film. As expected, no significant differences were observed in the thickness or morphology of the Al–O layer, so its contribution to the mechanical response can be considered constant throughout the study.

The known differences in deformation characteristics between the crystalline and amorphous phases are also evident in Figures 3 and 4. These observations are reflected not only in the cracks within the deformed amorphous layer but also in the deformation behavior of the substrate. Figure 3 clearly shows that the amorphous thin film exhibits a higher resistance to deformation, with most of the plastic deformation occurring in the substrate, as indicated by the green arrow pointing to the severely deformed Al region beneath the indentation imprint.

In contrast, the crystalline thin film accommodates more of the plastic deformation itself and transfers it to the Al substrate to a lesser extent. Furthermore, because of the plasticity of the crystalline Mo oxide layer, the deformation is transferred more uniformly to the substrate. In Figure 4, the cyan-colored region highlights the plastically deformed area within the substrate, which is thinner but present across the entire indentation perimeter.

During deformation, no signs of insufficient mechanical bonding between the coatings and substrate were observed.

### Mechanical Properties of the Nonlithiated State

In the following section, we present only the indentation experiments conducted up to 4 mN, as all tests performed at 6 mN led to the same conclusions (Supporting Information:Figures S2 and S3). To enable cross-validation with TEM investigations, we first examine the nonlithiated state. The data presented in Figure 7 are derived from the load–displacement curves of the indentation experiments shown in Figure 8. It is important to note that, based on the overview TEM images shown in Figures 3 and 4, we did not observe any measurable variation in film thickness, regardless of whether the calculation was performed near the indentation pattern or away from it, and this observation was independent of the phase of the deposited layer. Given that the residual deformation depths (*h*_rem_) extracted from the indentation curves are dominantly deeper than 200 nm, and the thickness calculated from the TEM images has a standard deviation of less than 10 nm due to surface roughness, we conclude that over 95% of the *h*_rem_ values originate from the substrate. These findings indicate that, in our coating–substrate system, the plastic deformation under the 10 μm spherical indenter is largely transferred to the substrate, irrespective of the phase of the coating. However, it should be emphasized that the *mode* of deformation transfer is not phase-independent. According to our TEM observations, in the crystalline phase, the plastic deformation is transferred across a broader lateral area and at shallower depths, while in the amorphous phase, it is transferred more locally and at greater depths.

**Figure 7:**
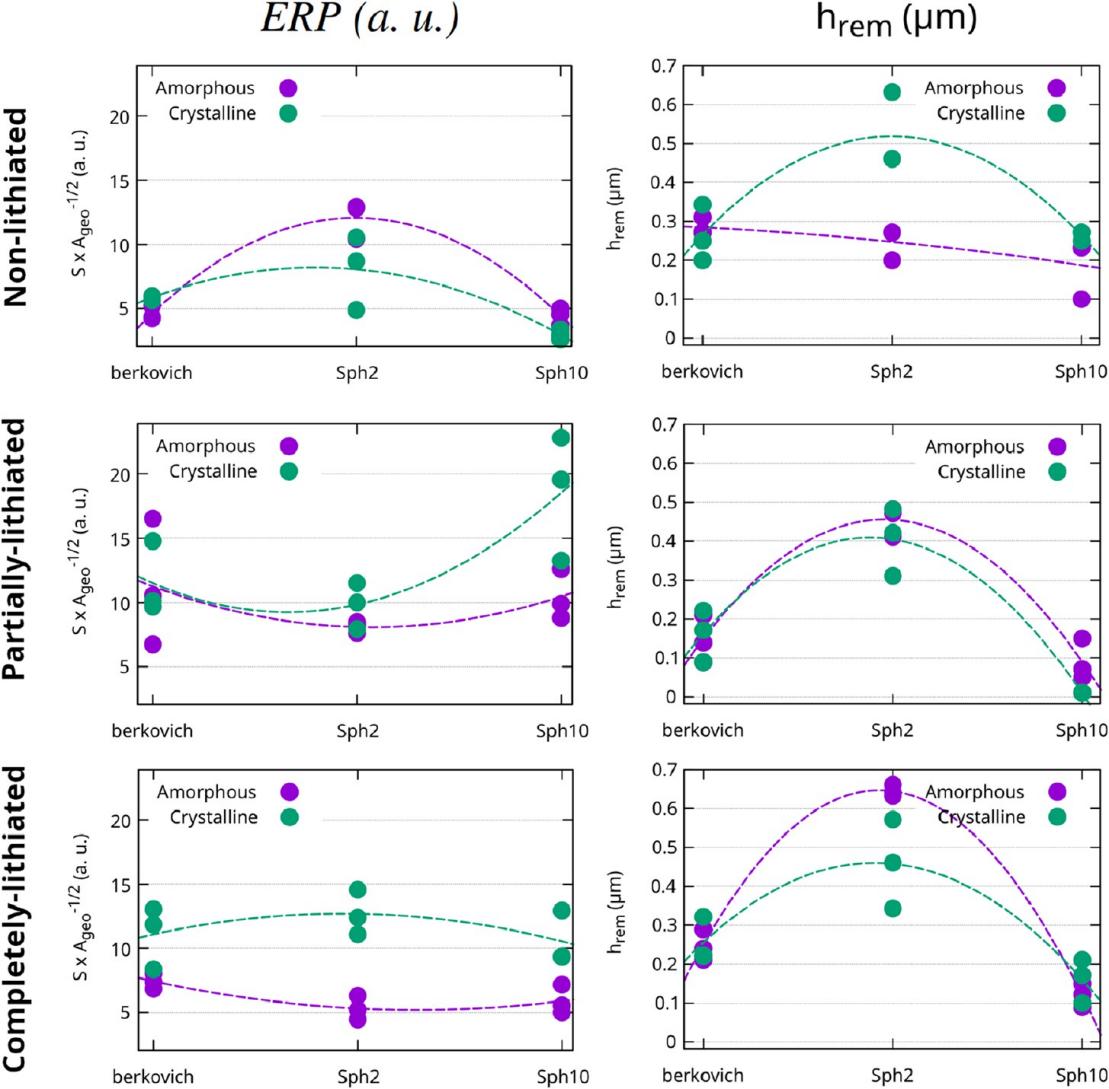
Mechanical properties derived from the load–displacement curves measured at a load of 4 mN (see Figure 8). Data for the amorphous layer are shown in purple, and for the crystalline layer in green. Each row corresponds to a different degree of lithiation. The *y*-axes in the left column represent the elastic response parameter of the system, characterized by the ERP = S/√*A*_geo_, while the right column shows the residual depth *h*_rem_, indicative of plastic properties. Each subplot presents the data as a function of the sharpness of the indenter tips.

**Figure 8:**
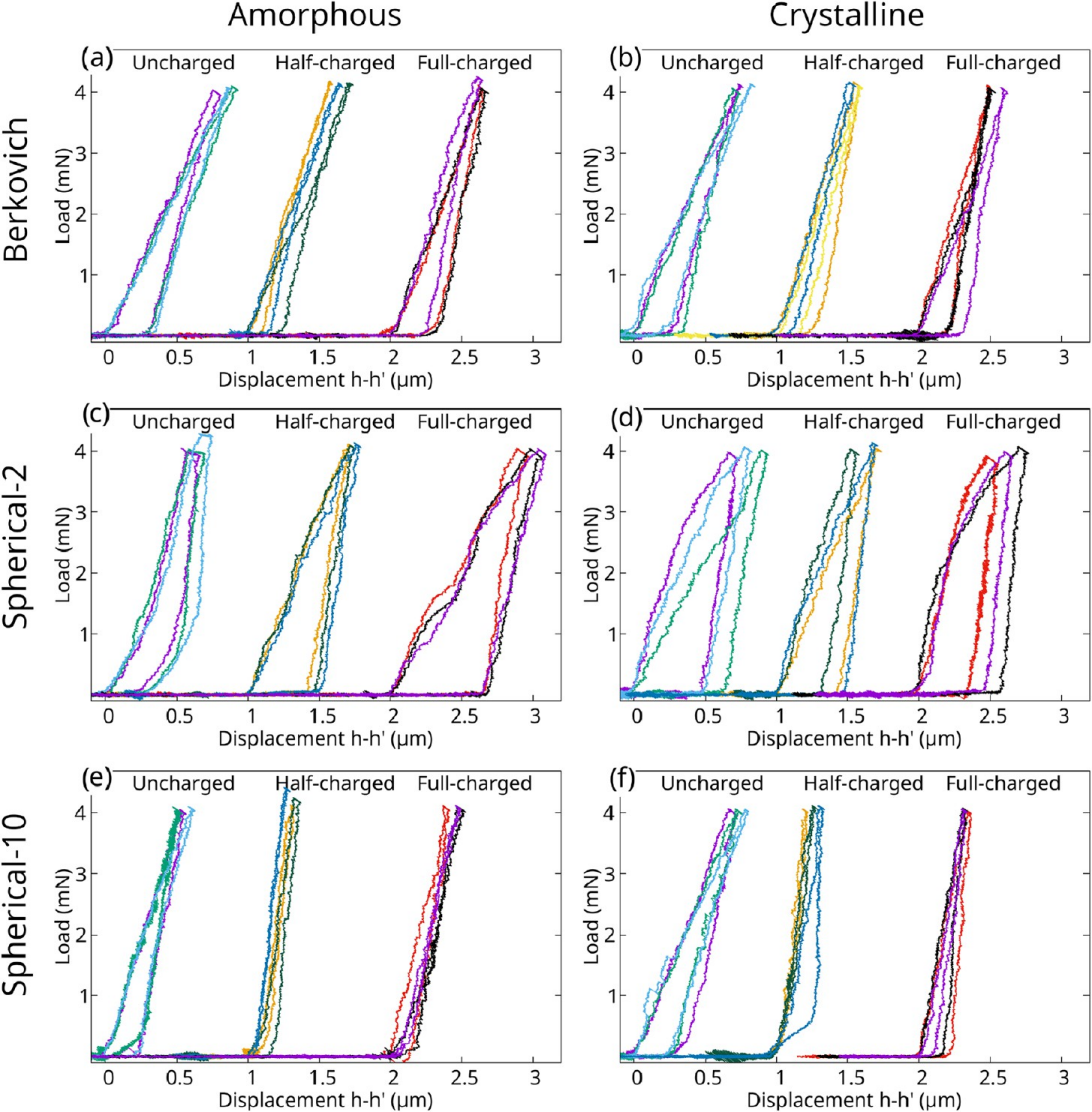
Load–displacement curves measured up to a 4 mN load, grouped in sets of three (same experimental conditions) and shifted along the *x*-axis by 0, 1, and 2 μm according to the state of charge (uncharged, half-charged, and fully charged) for better visualization. Left column: Curves measured on amorphous samples (a, c, e), while the right column shows the corresponding curves for crystalline samples (b, d, f). Rows correspond to the different indenter tip geometries (Berkovich (a, b), spherical-2 (c, d), and spherical-10 (e, f)).

When the Berkovich indenter was used, only limited phase-dependent differences were observed (Figure 7, upper row), although subtle variations in the extracted mechanical parameters are still present. Accordingly, the indentation curves exhibited similar characteristics (Figure 8). This reduced sensitivity can be attributed to the highly localized deformation imposed by the sharp Berkovich tip, which promotes an early onset of plasticity and results in a response dominated by irreversible deformation processes and substrate effects. In our previous work, under similar measurement conditions, the mechanical parameters derived from the indentation curves showed a clear dependence on the phase of the thin film.^3^ However, those measurements were performed on different substrates and with a different film thickness and are therefore not directly comparable to the current layer–substrate system.

The similarities of the mechanical response to Berkovich indentation can also be explained by the comparable plastic behavior of the different phases. The vertically columnar structure of the crystalline phase, as shown in Figure 5, may allow the Berkovich tip shape to accommodate grain boundary sliding. In this deformation mode, irreversible local changes occur in the amorphous regions directly adjacent to grain boundaries.^46^ This deformation mechanism may transmit mechanical stress in a manner characteristic of fully amorphous materials,^47^ further reducing the apparent differences between the two phases.

During spherical indentation, as expected, higher elastic response—proportional to the system’s elastic moduli—was measured for the amorphous layer. Correspondingly, the residual deformation (*h*_rem_), which characterizes plastic deformation, was lower in the case of the amorphous phase. For the crystalline phase, significant differences were observed only in measurements performed with the 2 μm radius spherical indenter. In this case, the residual indentation depth increased substantially, a trend also observable in the elastic responses.

### Effect of Lithium Intercalation on the Mechanical Properties

The intercalation of Li^+^ into a transition-metal oxide induces mechanical stresses, which in turn influence the mechanical and chemical properties of the entire ASSB system. In our experiments, we also investigated how the material’s response to deformation changes as a function of the degree of lithiation. The collected indentation curves shown in Figure 8 clearly demonstrate that the mechanical properties of the substrate–layer system depend on the amount of lithium intercalated into the layer. Each subplot in Figure 8 contains 3 × 3 curves, where sets of three curves were measured under identical conditions. The consistency of the load–displacement curves in a set indicates good reproducibility of the measurements. The degree of lithiation differs between these triplet groups, which significantly affects the shape and characteristics of the curves. These interpretations are based on electrochemically defined lithiation states established through controlled voltage cutoffs.

On a global scale—regardless of indenter geometry and microstructural phase—the elastic responses exhibit a consistent trend (ERP global averages: Non-Lith.: 6.3 ± 3.3; Part.-Lith.: 11.6 ± 4.4; Comp.-Lith.: 8.8 ± 3.2): the modulus increases in the presence of lithium. This indicates that a greater force is required to achieve the same degree of elastic compression when Li is present. While the phenomenon appears to be generally valid, it is most pronounced when using the 10 μm radius spherical indenter, which is the most sensitive to changes in elastic response. Furthermore, it can be concluded that although the dependence of the elastic modulus on lithium content is similar in both phases, the crystalline phase exhibits a higher sensitivity to the presence of Li, which suggests differences like lithium-hosting sites between the crystalline and amorphous phases.

Validating the applied measurement methodology, the observed plastic properties exhibit trends that are generally opposite to those of the elastic response, yet they also clearly reveal the presence of lithium. As confirmed by TEM investigations (nonlithiated sample, 10 μm spherical tip), the substrate plays a decisive role in governing plastic behavior. This effect is further supported by the larger differences observed when using the 2 μm spherical indenter tip. In this case, even the indentation response of the substrate without coating differed noticeably from those obtained with other tips.

The distinct mechanical response observed at partial lithiation can be interpreted in terms of microstructural heterogeneity arising during the lithium insertion. While the nonlithiated and fully lithiated states can be considered as relatively homogeneous configurations, the partially lithiated state represents a transient condition in which lithium distribution is spatially nonuniform.^23^ This results in locally varying bonding environments, oxidation states, and internal stress fields within the material. Such heterogeneity may lead to the coexistence of regions with different mechanical responses, effectively constraining deformation and increasing the resistance to both elastic and plastic strain. As a result, the partially lithiated state exhibits an apparent increase in the ERP and a reduction in residual deformation. This interpretation is consistent with recent studies reporting heterogeneous lithiation pathways and spatially localized structural evolution in transition-metal oxides.

The residual deformation (*h*_rem_), which characterizes plastic properties, also exhibits a strong dependence on the Li^+^ content. Regardless of the phase or indenter geometry, the smallest residual depths were consistently observed in the partially lithiated state. This further supports the notion that nonlithiated and fully lithiated systems represent distinct but internally consistent mechanical states, a conclusion that also holds true for the amorphous phase.

## Conclusions

We investigated the cathode–current collector system of Li-ion batteries, specifically focusing on MoO_3_ thin film cathodes and aluminum substrates, using nanoindentation techniques. Both elastic and plastic properties were characterized using indenters of varying geometries, while the layer–substrate mechanical interaction was examined by penetration depths comparable to the coating thickness. To examine the effect of lithium intercalation into the cathode, measurements were performed under inert conditions on fully and partially lithiated samples as well.

In our experiments, no strain bursts were observed in the presence of the coating in contrast to the indentation with a 2 μm spherical tip on the bare substrate. As the majority of plastic deformation occurs in the substrate, it is assumed that (a) the well-adhering coatings (both crystalline and amorphous) transmit mechanical strain in a manner that alters the effective tip geometry perceived by the substrate; and (b) the newly formed interface, distinct from a free surface, may enable or inhibit dislocation nucleation/annihilation, thereby influencing dislocation dynamics and suppressing strain bursts. This suppression effect is consistent with literature findings where additional grain boundaries produced a similar outcome.^48^

In the case of the nonlithiated states, most of the plastic deformation was found to take place within the substrate, with the amorphous phase transmitting this deformation more locally. This may be attributed to the higher EPR parameter of the amorphous layer (Figure 7, top row). Due to its increased stiffness and brittleness, the amorphous layer tends to crack along the indenter’s contact edge (Figures 2 and 3). As a result, mechanical response becomes concentrated within the cracked contact zone. In contrast, the crystalline layer—due to its plasticity—more uniformly transfers the load to the substrate and can accommodate the indenter’s shape through grain boundary sliding during loading.

The results obtained using the Berkovich tip differ fundamentally from those with the spherical indenters, as the geometry of the Berkovich tip emphasizes plastic properties even in the early stages of indentation. On a global scale, Berkovich indentation revealed no significant mechanical differences with respect to either the coating phase or lithium content, suggesting a similar plastic deformation mechanism throughout the substrate–layer system. In the crystalline phase, deformation occurs primarily along grain boundaries. Based on this observation, it is reasonable to consider that lithium intercalation may not be restricted solely to the grain interiors, and grain boundary regions could also contribute to lithium accommodation. In particular, amorphous regions associated with grain boundaries may provide energetically accessible sites for lithium uptake. However, this interpretation is based on indirect mechanical evidence, and no direct chemical or spatially resolved measurements were performed to verify lithium distribution within specific microstructural features.

The elastic properties of the layer–substrate system were found to be strongly dependent on the amount of lithium stored within the cathode. In addition to demonstrating the mechanical influence of the thin film on deformation response, this result also confirms a strong mechanical coupling between the layer and substrate. Notably, the partially lithiated system exhibited the highest ERP parameter, which can be attributed to the increased microstructural heterogeneity arising from nonuniform lithium distribution. In contrast to the nonlithiated and fully lithiated states, which represent more homogeneous configurations, the partially lithiated system contains spatial variations in local structure and stress state, leading to an effectively enhanced resistance to deformation.

Average values of residual deformation (*h*_rem_) were lowest in the partially lithiated state, which can be attributed to the presence of microstructural heterogeneity and spatially varying deformation resistance. The nonuniform lithium distribution may limit the activation of localized plastic deformation mechanisms while promoting a more constrained deformation field. Similarly, in amorphous materials, plastic deformation occurs through mechanisms associated with free volume.^47^ The gradual intercalation of lithium reduces these available free volume sites, whereas the fully lithiated state may form a uniform, distinct phase in which the effective free volume contributing to deformation is higher than in the partially lithiated state.

## Supplementary Material



## Notes

First-cycle voltage–capacity curves of amorphous (AS) and heat-treated (HT) MoO_3_ thin film electrodes at different lithiation states; mechanical properties derived from the load-displacement curves measured at a load of 6 mN; load–displacement curves measured up to a 6 mN load, grouped in sets of three (same experimental conditions) and shifted along the x-axis by 0, 1, and 2 μm according to the state of charge (uncharged, half-charged, and full-charged) for better visualization (PDF)
