# Supplementary material for: Lithiation-Dependent Micromechanical Response of Amorphous and Crystalline MoO3 Thin-Film Cathodes on Al Current Collectors
Source: ACS Omega. 2026 Jun 25;11(27):39841–52. doi: 10.1021/acsomega.6c00534 (PMC13382683; doi:10.1021/acsomega.6c00534)
Supplement: Supplementary file 1 [file ao6c00534_si_001.pdf]

SUPPLEMENTARY INFORMATION

Lithiation-dependent micromechanical response of amorphous and crystalline MoO_3 thin film cathodes on Al current collectors

*Dávid Ugi [1,2], Lakshmi Shiva Shankar [3], G. Z. Radnóczi [4], Péter Dusán Ispánovity [2,5], Robert Kun [1,6]**

[1] HUN-REN Research Centre for Natural Sciences, Institute of Materials and Environmental Chemistry, Magyar Tudósok Körútja 2, 1117 Budapest, Hungary

[2] ELTE Eötvös Loránd University, Department of Materials Physics, Pázmány Péter sétány 1/a, 1117 Budapest, Hungary

[3] Széchenyi István University, Zalaegerszeg Innovation Park, Dr. Michelberger Pál út 3, H-8900 Zalaegerszeg, Hungary

[4] HUN-REN Centre for Energy Research, Konkoly-Thege M. u. 29-33, Budapest H-1121, Hungary

[5] HUN-REN Wigner Research Centre for Physics, Institute for Solid State Physics and Optics, Konkoly T. út 29-33., Budapest, 1525, Hungary

[6] Széchenyi István University, Sustainability Competence Centre, Egyetem square 1., H-9026 Győr, Hungary

KEYWORDS: Solid state Li-ion batteries; Lithium intercalation; In situ nanoindentation; MoO3 thin film

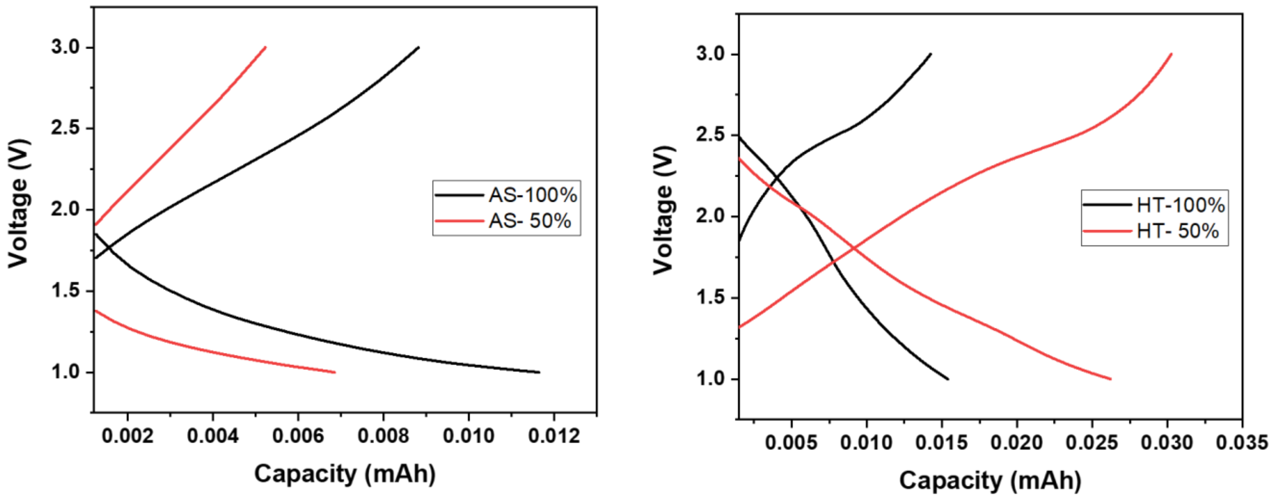

Figure S1. First-cycle voltage–capacity curves of amorphous (AS) and heat-treated (HT) MoO_3 thin film electrodes at different lithiation states.

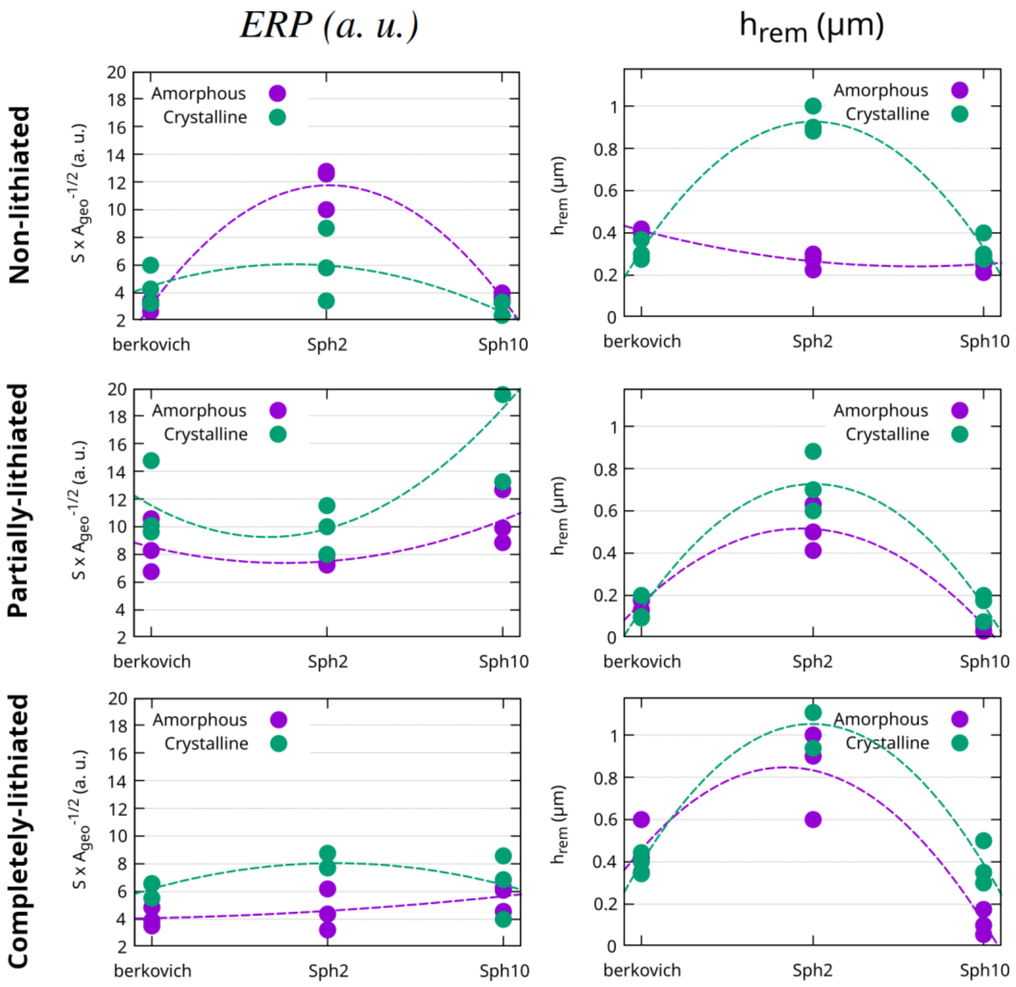

Figure S2. *Mechanical properties derived from the load-displacement curves measured at a load of 6 mN (see Figure xy). Data for the amorphous layer are shown in purple, and for the crystalline layer in green. Each row corresponds to a different degree of lithi ation. The y-axes on the left column represent the elastic response paremeter of the system, characterized by the ERP =* $S/\sqrt{A_{geo}}$*, while the right column shows the residual depth* h_{rem}*, indicative of plastic properties. Each subplot presents the data as a function of the sharpness of the indenter tips.*

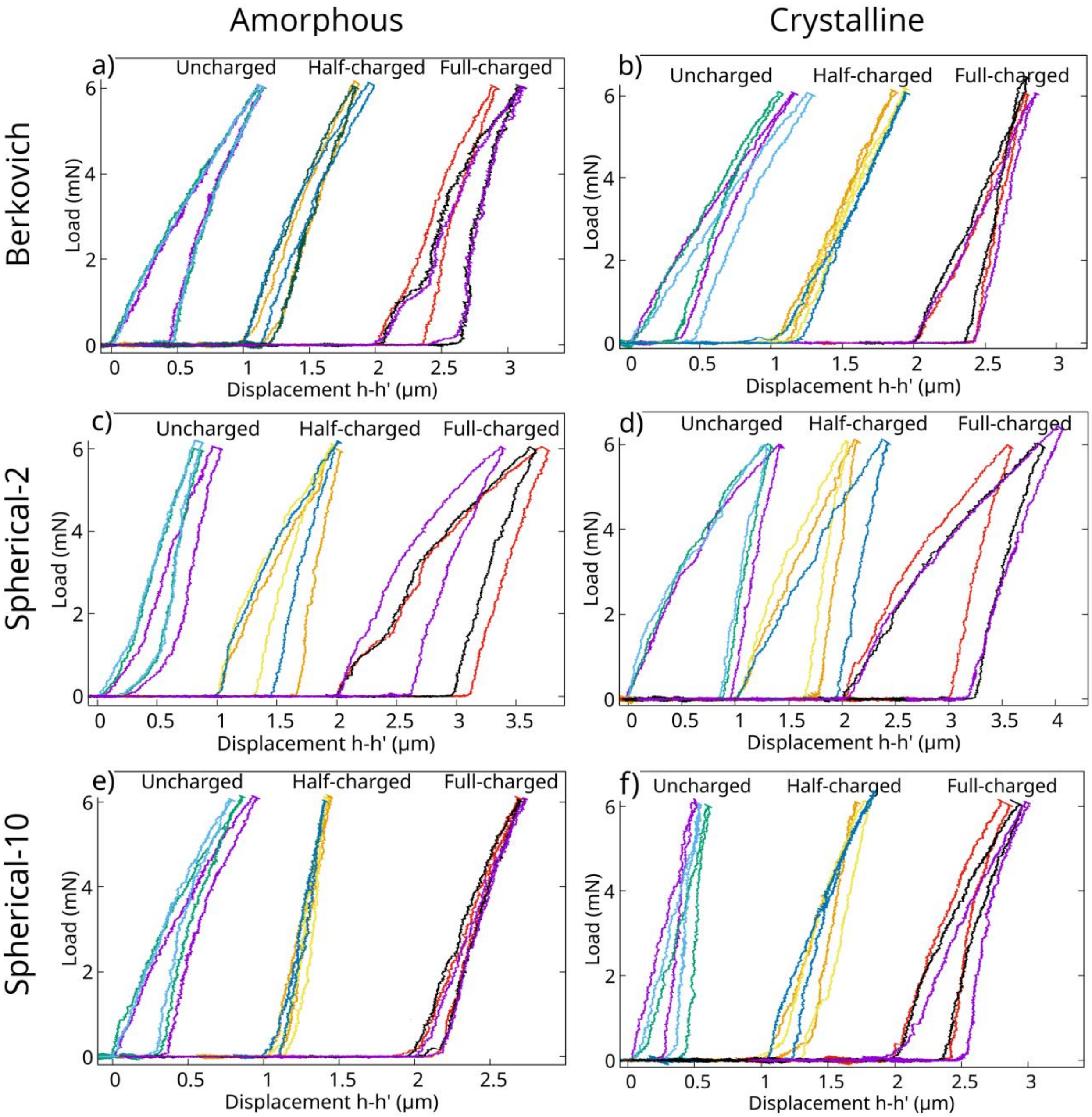

Figure S3. Load–displacement curves measured up to a 6 mN load, grouped in sets of three (same experimental conditions) and shifted along the x-axis by 0, 1, and 2 µm according to the state of charge (uncharged, half-charged, and full-charged) for better visualization. Left column: Curves measured on amorphous samples, while the right column shows the corresponding curves for crystalline samples. Rows correspond to the different indenter tip geometries (Berkovich, Spherical-2, and Spherical-10).
